# Comparative proteomic analysis of spermatozoa isolated by swim-up or density gradient centrifugation

**DOI:** 10.1186/s12958-015-0027-y

**Published:** 2015-04-19

**Authors:** Stefania Luppi, Monica Martinelli, Elisa Giacomini, Elena Giolo, Gabriella Zito, Rodolfo C Garcia, Giuseppe Ricci

**Affiliations:** Institute for Maternal and Child Health, IRCCS Burlo Garofolo, Via dell’Istria 65/1, 34137 Trieste, Italy; Department of Medicine, Surgery and Health Sciences, University of Trieste, 34149 Trieste, Italy; International Centre for Genetic Engineering and Biotechnology (I.C.G.E.B.), Area Science Park, 34149 Trieste, Italy

**Keywords:** Density gradient centrifugation, Sperm cells, Swim-up, Protein 2-dimensional maps

## Abstract

**Background:**

Reports about the morphologic and functional characteristics of spermatozoa prepared by density gradient centrifugation (DC) or swim-up (SU) have produced discordant results. We have performed a proteomic comparison of cells prepared by DC and SU providing a molecular insight into the differences between these two methods of sperm cell isolation.

**Methods:**

Protein maps were obtained by 2-dimensional (2-D) separations consisting of isoelectrofocusing (IEF) from pI 3 to 11 followed by SDS-polyacrylamide gel electrophoresis. 2-D gels were stained with Sypro Ruby. Map images of DC and SU spermatozoa were compared using dedicated software. Intensities of a given spot were considered different between DC and SU when their group mean differed by >1.5-fold (p < 0.05, Anova).

**Results:**

No differences were observed for 853 spots, indicating a 98.7% similarity between DC and SU. Five spots were DC > SU and 1 was SU > DC. Proteins present in 3 of the differential spots could be identified. One DC > SU spot contained lactate dehydrogenase C and gamma-glutamylhydrolase, a second DC > SU spot contained fumarate hydratase and glyceraldehyde-3-phosphate dehydrogenase-2, and a SU > DC spot contained pyruvate kinase M1/M2.

**Conclusions:**

The differences in protein levels found on comparison of DC with SU spermatozoa indicate possible dissimilarities in their glycolytic metabolism and DNA methylation and suggest that DC cells may have a better capacitation potential.

**Electronic supplementary material:**

The online version of this article (doi:10.1186/s12958-015-0027-y) contains supplementary material, which is available to authorized users.

## Background

Sperm quality is crucial to assisted reproductive techniques [1]. Regarding the two most conventional methods of sperm separation, SU and DC, recovery rates of total motile, progressive motile and viable sperm cells have been shown to be higher after DC than after SU [2,3]. Instead, Chantler *et al*. [4] observed that the proportion of fast spermatozoa was enhanced in SU preparations. Always comparing DC and SU, Monqaut *et al*. [5] reported less vacuolization in SU and both Prakash *et al*. [6] and Hammadeh *et al*. [7] found a higher percentage of morphologically normal spermatozoa after DC. Xue *et al*. [8] reported a lower deformity rate and DNA fragmentation index after DC. According to Fraczek *et al.* [9], spermatozoa selected by SU show a slightly better viability and morphology than cells isolated by DC and a much higher capacity to inhibit the secretion of reactive oxygen intermediates (ROIs) by stimulated leukocytes. Other authors state that ROIs produced during the SU procedure can have detrimental effects on their viability, motility, membrane function and penetration ability [10-12]. It has been postulated that the SU technique may not be convenient to isolate sperm cells from ejaculates showing a high level of ROI production [13]. Fertilization rates have been observed to be either the same for DC and SU [14] or higher for DC [15,16]. Pregnancy rates for SU against DC were reported to be 46.2% and 57.1% respectively for intra-cytoplasmic fertilization [17]. After in-vitro sperm-egg fertilization, pregnancy rates using SU or DC cells were reported to be, respectively, 21.1% and 33.3% [18] or 33.3% and 32.8% [7].

Protein fingerprinting affords an evaluation of cells at molecular level. Cell protein compositions can be compared by analyzing 2-dimensional protein spot maps obtained by isoelectric focusing (IEF) followed by dodecyl sulfate gel electrophoresis (SDS-PAGE) [19,20]. IEF separates proteins on gel strips according to their isoelectric point (pI), while SDS-PAGE resolves molecules as a function of their molecular mass. The use of the protein dye Sypro Ruby [21] and the optimization of Coomassie blue staining [22] have improved spot detection sensitivity and quantification ranges. Approximately 1,000 protein spots can be visualized in a 2-D separation. Spot excision, in-gel proteolysis and mass spectrometry results in protein identifications [23]. A crucial feature of 2-D techniques is the detection of post-translational modifications such as phosphorylation, glycosylation, proteolytic cleavages, etc., which are often related to changes in function. Comparative proteomic analyses of sperm cells have been performed in relationship with different features such as capacitation [24], motility [25,26], globozoospermia [27], semen oxidative stress [28,29], poor blastocyte development after intra-cytoplasmic sperm injection [30] and lack of binding to the zona pellucida [31], all of which influence fertility. To date, no studies have compared the proteomic profiles of spermatozoa prepared by SU or DC. We report here such an analysis, covering the pI range 3–11 and molecular masses from 175 to 6 kDa.

## Methods

This study was conducted from 2009 till 2011 after approval by the Institutional Review Board of the Institute for Maternal and Child Health IRCCS Burlo Garofolo, Trieste, Italy (RC 35/08), in agreement with the WMA Declaration of Helsinki about Ethical Principles for Medical Research. Signed informed consent was obtained from each participant of this study.

### Sperm cells preparation

Semen samples from 4 normozoospermic Caucasian subjects (mean age 34.8 yrs, range 22–44) were processed according to World Health Organization guidelines [32] as previously described [3]. Spermatozoa were prepared by swim-up (SU) or density gradient centrifugation (DC) [3,33]. Briefly, SU cells were obtained from the upper layer after layering medium containing 0.5% human serum albumin at a 45° angle on a suspension of washed sperm cells and incubating at 37°C for 45 min. DC cells consisted of the resuspended pellet obtained after loading liquefied semen on a 40-80% double density gradient (PureSperm, Nidacon International, AB, Goteborg, Sweden) and centrifuging at 300 × *g* for 20 min. Motilities of whole semen and cells prepared by DC and SU were determined (n = 4).

### Protein isolation and separation

Sperm cells (3 × 10^6^) were washed twice with 9% (v/v) sucrose and solubilized with 250 μl of DeStreak® rehydration solution (GE Healthcare, Uppsala, Sweden) with the addition of 1% (v/v) IPG 3–11 solution (GE Healthcare, Uppsala, Sweden). This solubilization mixture contains optimized concentrations of urea, thiourea, CHAPS detergent, DeStreak® Reagent and ampholytes. It is a strong protein solubilizer that also prevents protein streaking and oxidation during the isoelectrophoretic run. Samples were processed for IEF by sonicating at amplitude 10 μm for 5 sec followed by centrifugation at 10 000 × *g* for 3 min, as previously described [34]. The particle-free supernatants after sonication were used for in-gel swelling of 13 cm long IEF strips pI 3–11 (GE Healthcare, Uppsala, Sweden). Focusing was performed on a Protean II IEF cell (BioRad, Hercules, CA, USA) up to 48 000 VXhr. Focused proteins were separated by placing the IEF strips on 14% SDS-PAGE gels (W160XL140X1.5 mm) along with 6–175 kDa markers. Electrophoresis was conducted so as to keep within gels proteins of molecular mass down to 6 kDa.

### Protein visualization and calculation of parameters

Gels were fixed for 1 h in 10% (v/v) ethanol-7% (v/v) acetic acid, rinsed with water and stained with Sypro Ruby (Invitrogen, Eugene, OR, USA) for 1 day. Background fluorescence was washed off and Sypro Ruby images were analyzed as described below. Imaged gels were washed with water, stained with Colloidal G-250 Coomassie Blue [22] and scanned at 300 dpi in an Epson Expression 1680 Pro (Epson, Long Beach, CA, USA).

Isoelectric point values were calculated according to the pI curve of 3–11 non-linear Immobiline DryStrip gels [35]. Experimental molecular masses were calculated from semi-logarithmic (log_10_) curves of molecular mass vs. migration distance.

### Image analysis

Sypro Ruby-stained gel images were analyzed at the Ludesi Analysis Center (Lund, Sweden, http://www.ludesi.com) using the Redfin 3 software. All-to-all-spot gel matching avoided the bias of reference gels. The cumulative staining intensity of each spot is referred to as volume. Spot intensities were background and noise corrected. Gels were normalized according to their total protein content taken as the sum of all spot intensities. Normalization made spot volumes comparable between gel images, eliminating differences from staining, protein loading and/or scanning velocity. A comprehensive total of 864 spots per gel were detected. Proteins were considered as differentially expressed when the mean spot intensity differed by > 1.5-fold (p < 0.05, Anova) on comparison of SU with DC.

### Protein identification by mass spectrometry

Protein spots showing statistically significant differences in intensity between DC and SU were excised from Coomassie-stained gels, digested with trypsin and identified by nano LC-ESI-MS/MS by Proteome Factory AG (Berlin, Germany). An Agilent 1100 nanoLC system (Agilent, Santa Clara, CA, USA) coupled to an ABI Q-Star XL Q-TOF mass spectrometer (Applied Biosystems, Foster City, CA, USA) were used. The search engine was Mascot (Matrix Science, Boston, MA, USA). Selection filters were: MOWSE (Molecular Weight Search) score ≥ 70, peptide coverage ≥ 10% and an acceptable correspondence between experimental and theoretical molecular masses and pI values.

## Results

### Sperm cell parameters

All samples were normospermic (152 × 10^6^/mL ± 86 × 10^6^/mL), of progressive motility 64.8% ± 15.2% (means ± SD, n = 4), normal pH and viscosity, and contained 0.04 × 10^6^/mL ± 0.07 × 10^6^/mL leucocytes based on the standard peroxidase method [32] and 4.7 × 10^6^/mL ± 7.2 × 10^6^/mL round cells (means ± SD, n = 4). Progressive motility was 77.8% ± 3.9% after DC and 87.5% ± 9.6% after SU sperm preparation (means ± SD, n = 4), the difference not being significant (Anova, paired, no replicates).

### 2-dimensional protein separations and comparative analysis

**Figure 1:**
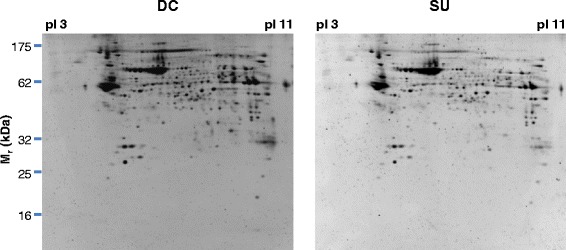
2-dimensional separation of sperm cell proteins. Representative protein maps of Sypro Ruby-stained gels corresponding to one subject. Sperm cells were obtained by DC or SU. Isoelectric focusing covered the range pI 3- pI 11. Molecular masses (M_r_) are indicated on the left axis.

**Figure 2:**
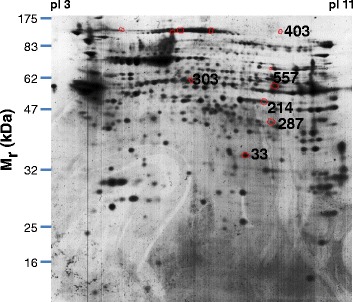
Location of spots differing ≥1.5-fold in their intensity on comparison of DC with SU cells. Sypro Ruby-stained gels were stained with colloidal Coomassie Blue for quantitative analysis. A representative protein map shows encircled in red the 11 spots for which significant differences ≥1.5-fold (p < 0.05, Anova) were found between DC and SU group. The differences are either DC > SU or SU > DC, as indicated in Figure 3 and Table 1. The 6 spots that are numbered were considered to be reliable because located in areas of the gels not affected by horizontal streaking and/or a strong background.

**Figure 3:**
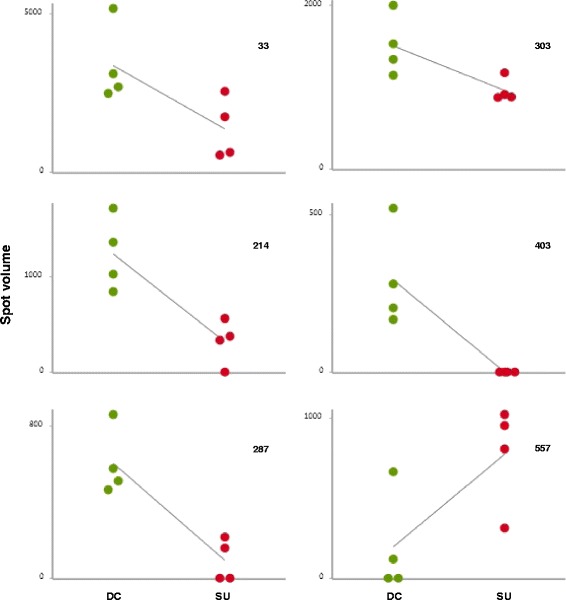
Quantitative analysis of spot intensities in DC compared with SU. Comparative dot plots of spot intensities (volume = intensity) corresponding to DC (green dots) and SU (red dots). Intensities are from images of Sypro Ruby-stained gels normalized and quantified using the Ludesi Redfin 3 software.

Spermatozoal proteins from DC or SU cells were separated by IEF followed by SDS-PAGE and spots were visualized by Sypro Ruby staining (Figure 1) and image analyzed using the Redfin 3 software as explained in detail in Methods. The mean intensity of 853/864 spots (98.7% of the total) differed by <1.5-fold (p < 0.05, Anova) on comparison of DC with SU. Differences ≥1.5-fold were observed for 11 spots, with 5 being unreliable due to horizontal streaking and/or strong background (Figure 2). Five of the 6 reliable spots (#33, #214, #287, #303 and #403) were 2.5- to 3.9-fold more intense in DC than in SU, while spot #557 was 3.8-fold stronger in SU (Figure 3, Table 1).

**Table 1:** **Proteins differentially expressed in spermatozoa prepared by DC with respect to SU**

**Spot #**	**Protein identity**	**UniProtK accession number**	***M***_**r**_ **/p** ***I*** **theor.**	***M***_**r**_ **/p** ***I*** **exper.**	**Seq. cover.**	**Nr. pept.**	**Score**	**DC/SU fold change**
**33**	Lactate dehydrogenase, C chain	P07864	36.3/7.1	34.7/7.0	39%	15	811	2.47
**33**	γ-Glutamylhydrolase	O92820	33.6/7.2	34.7/7.0	19%	5	168	2.47
**214**	Fumarate hydratase	P07954	50.0/7.0	51.3/7.8	39%	22	805	3.90
**214**	Glyceraldehyde-3-P dehydrogenase 2/S	O14556	44.5/8.4	51.3/7.8	23%	9	226	3.90
**557**	Pyruvate kinase M1/M2	P14618	57.8/7.9	69.2/7.9	35%	20	673	0.26

Theor.: Theoretical; Exper.: Experimental; Nr. Pept.: Number of matched peptides; Score: MOWSE score.

See Additional file 1, Supplementary information.

### Protein identifications

Differential spots were excised from gels. Nano LC-ESI-MS/MS of the tryptic peptides obtained from each spot led to the identification of the following proteins: lactate dehydrogenase chain C (LDH-C, spot #33), γ-glutamyl hydrolase (γ-GH, spot #33), fumarate hydratase (FH, spot #214), glyceraldehyde-3-phosphate dehydrogenase-2 (GAPDH-2, spot #214) and pyruvate kinase M1/M2 (PKM, spot #557) (Table 1 and Additional file 1, Supplementary information). Spots 33 and 214 contained 2 co-migrating proteins each, while spot 557 contained one protein. The remaining 3 spots did not yield results. It is not possible to establish the precise contribution of each of the co-migrating proteins to the differences in intensity between DC and SU observed for spots 33 and 214.

The experimental pIs of LDH-C, γ-GH and PKM were in good agreement with the theoretical ones, whereas those of FH and GADPH-2 differed by + 0.8 and −0.6 units from the theoretical values, respectively (Table 1). The experimental molecular masses of LDH-C, γ-GH and FH were within ± 3.5% (mean, SD = 1.1) of their theoretical values. Instead, the experimental molecular masses of GADPH-2 and PKM were 15.3% and 19.7% above their theoretical values, respectively (Table 1).

## Discussion

Our work compared for the first time the 2-D protein pattern of sperm cells obtained by DC and SU. We visualized protein spots with the sensitive stain Sypro Ruby [21] and obtained maps from 6 to 175 kDa and pI 3 to 11, a wider range than that of published sperm cell 2-D maps [36-39]. 2-D comparative analysis offers advantages such as the visualization of post-translational modifications (PTMs) and proteolytic fragments as well as protein level quantifications based on the intensity of each spot, as opposed to spectral counts of peptides. The latter is an important consideration because peptides could originate in unmodified, post-translationally modified or fragmented proteins. The protein composition of sperm cells provides information regarding cell function, therefore is closely linked to fertility [40]. Membrane proteins are involved in capacitation, binding to the oocyte and the subsequent acrosome reaction. Acrosomal proteins participate in the process of oocyte penetration. Nuclear proteins are relevant to the chromatin condensation state. Mitochondrial proteins, concentrated in the midpiece, are central regarding energy metabolism. Tail proteins are crucial for sperm cell progressive movement. Cytosolic proteins involved in metabolism and glycolytic enzymes of the fibrous sheath are also essential. Protein expression levels are therefore related to cell functions and fertilization ability.

The comparison of DC with SU cells we performed showed no statistically significant spot intensity differences ≤ 1.5-fold (p < 0.05) for 98.7% of the total number of spots. This suggests a strong similarity in the protein composition of sperm cells prepared by either of these 2 methods. The intensity of 5 spots was significantly stronger in DC while one spot was more intense in SU. Four of the proteins overexpressed in DC cells were identified as lactate dehydrogenase chain C (LDH-C), γ-glutamyl hydrolase (γ-GH), fumarate hydratase (FH) and glyceraldehyde-3-phosphate dehydrogenase-2 (GAPDH-2). Instead, pyruvate kinase M1/M2 (PKM) levels were higher in SU cells. No stress-related proteins were found.

LDH-C, FH, GADPH-2 and PKM have been reported in the human sperm cell proteome [37-39,24]. LDH-C and GADPH-2 are unique to sperm cells [41,42]. FH, γ-GH, GAPDH-2 and PKM have been described as targets of S-nitrosylation in human spermatozoa isolated by DC [43]. GADPH-2, PKM and LDH are enzymes of the glycolysis pathway [44].

The differences observed between experimental and theoretical pIs observed for FH and GADPH-2 could be due to PTMs that either eliminate or create charges. FH contains 10% of acidic amino-acids liable to derivatization, which would result in an increase in pI. Alternatively, arginylation might have occurred [45]. GADPH-2 could have become more negatively charged e.g. due to the derivatization of basic aminoacids or by phosphorylation. In fact, addition of one phosphate group would result in a theoretical decrease of 0.64 pI units to pI 7.75 according to PhosphoSitePlus® (http://www.phosphosite.org/isoelectricCalcAction.do?id=24581&residues=20) [46], in agreement with the pI value of 7.8 we observed.

The fact that the experimental molecular masses of GADPH-2 and PKM were 15.3% and 19.7% above their theoretical values, respectively, could be an indication of PTMs or of an anomalous migration on SDS-PAGE. GADPH-2 has indeed been reported to contain a proline-rich stretch within its extra N-terminal portion conferring biochemical properties such as insolubility and a slow electrophoretic migration [42,47]. Slow migration manifests itself as an apparent greater molecular mass.

γ-GH is a lysosomal endo/exo-peptidase catalyzing the hydrolysis of polyglutamylated folate into monoglutamates. Polyglutamylated folates are substrates for several enzymes involved in the generation of the primary methyl group donor S-adenosylmethionine. Hence, γ-GH modulation may affect DNA methylation, which is an important epigenetic determinant in gene expression and maintenance of DNA integrity. In cancer cell lines, γ-GH over-expression has been reported to decrease global DNA methylation and DNA methyl-transferase activity [48].

FH participates in the mitochondrial tricarboxylic acid cycle. Since we detected a not yet reported FH form with a pI considerably higher than the theoretical value, no speculation on the role of the increase of this particular FH form in DC cells can be made.

The diminished levels of PKM we observed in DC cells could lead to a reduction in glycolysis because PKM acts at the last, rate-limiting step of this pathway. Glycolysis is the primary energy pathway for sperm metabolism and supplies ATP to the flagellar dynein ATPase motility system [49]. Since the motility of DC and SU cells was not observed to be significantly different, the lower PKM levels in DC cells may be unimportant at least in conditions of a satisfactory nutrient supply to sperm cells.

GADPH-2 is a glycolytic enzyme catalyzing the oxidative phosphorylation of glyceraldehyde-3-phosphate (GAL-3-P) to yield 1,3-diphosphoglycerate and NADH. 1,3-diphosphoglycerate is then used by phosphoglycerate kinase to produce ATP. Mice lacking GADPHS, the ortholog of human GADPH-2, show a 90% reduction in cellular ATP and profound defects in motility and infertility [50]. GADPH-2 is bound to the fibrous sheath of sperm flagella. Of note, an increase in GADPH-2 has been reported in reactive oxygen species-negative sperm cells [28] which, in relationship with our results, would be consistent with DC cells producing reduced amounts of oxygen metabolites compared with SU cells. A very recent report describes GADPH-2 as localized in the apical part of the sperm head as well as in the principal piece of the flagellum, suggesting a potential role of GAPDH-2 together with other proteins in the secondary or post-acrosome reaction binding of sperm cells to oocytes [47].

LDH-C was found over-expressed in DC cells. This enzyme could employ the NADH generated during GAL-3-P oxidation by GADPH-2 for the reduction of pyruvate to lactate. Interestingly, sperm ATP levels, motility, hyperactivation and tyrosine phosphorylation have been found to increase in the presence of exogenous pyruvate in combination with glucose. This led to the proposal that pyruvate may promote male fertility by enhancing the glycolytic flux through its conversion to lactate by LDH-C, resulting in an improved capacitation [51]. The higher level of LDH-C in DC cells compared with SU cells could assist such a scenario, which would be favourable to successful in-vitro fertilization outcomes.

## Conclusions

On the basis of the differences in protein levels observed comparing DC with SU cells, there could be dissimilarities in their glycolytic capacity and in DNA methylation. Capacitation and post-acrosome binding of DC cells are potentially more favourable.

## Additional file

Additional file 1:
**Supplementary information.** Supporting data on protein identifications: Mascot search.
