# Supplementary material for: Comparative proteomic analysis of spermatozoa isolated by swim-up or density gradient centrifugation
Source: Reprod Biol Endocrinol. 2015 Apr 19;13:36. doi: 10.1186/s12958-015-0027-y (PMC4410587; doi:10.1186/s12958-015-0027-y)
Supplement: Additional file 1: — Supplementary information. Supporting data on protein identifications: Mascot search. [file 12958_2015_27_MOESM1_ESM.pdf]

Additional file 1, Supplementary information
Supporting data on protein identifications: Mascot search

Spot #33
gi|4504973 Mass: 36288 Score: 811 Matches: 32
(15) Sequences: 18(11) emPAI: 2.71
L-lactate dehydrogenase C chain [Homo sapiens]
Check to include this hit in error tolerant search

Query	Observed	Mr(expt)	Mr(calc)	ppm	Miss	Score	Expect	Rank	Unique	Peptide
1	350.2243	698.4341	698.4439	-14.00	0	(42)	0.036	1	U	R.LALVQR.N
2	350.2259	698.4372	698.4439	-9.56	0	(33)	0.27	1	U	R.LALVQR.N
3	350.2277	698.4409	698.4439	-4.30	0	(29)	0.56	1	U	R.LALVQR.N
4	350.2351	698.4557	698.4439	16.9	0	43	0.019	1	U	R.LALVQR.N
5	350.2441	698.4737	698.4439	42.7	0	(12)	16	1	U	R.LALVQR.N
12	361.7110	721.4074	721.4010	8.80	0	34	0.22	1	U	R.YLIGEK.L
19	380.2147	758.4148	758.4174	-3.41	0	33	0.6	6		K.EQLIEK.L
20	380.2209	758.4272	758.4174	13.0	0	(30)	1	7		K.EQLIEK.L
38	409.2186	816.4226	816.4341	-14.20	0	62	0.00055	1	U	R.NGVSDVVK.I
39	409.2227	816.4309	816.4341	-4.02	0	(48)	0.014	1	U	R.NGVSDVVK.I
40	409.2228	816.4311	816.4341	-3.75	0	(43)	0.045	1	U	R.NGVSDVVK.I
48	421.7623	841.5100	841.5022	9.36	0	31	0.28	1	U	K.ISGLPVTR.V
49	421.7798	841.5451	841.5022	51.0	0	(8)	33	2	U	K.ISGLPVTR.V
66	450.2874	898.5602	898.5600	0.28	0	(40)	0.041	1	U	R.IVIVTAGAR.Q
67	450.2921	898.5696	898.5600	10.7	0	56	0.00072	1	U	R.IVIVTAGAR.Q
87	499.2698	996.5251	996.5426	-17.57	0	6	1.5e+02	2	U	R.VHPVSTMVK.G
88	499.7297	997.4449	997.4465	-1.59	0	45	0.01	1	U	K.DYSVSANSR.I
89	499.7318	997.4490	997.4465	2.56	0	(26)	0.91	1	U	K.DYSVSANSR.I
24	385.2263	1152.6572	1152.6438	11.7	1	30	0.71	1	U	R.RVHPVSTMVK.G
25	385.2431	1152.7074	1152.6438	55.2	1	(6)	1.1e+02	3	U	R.RVHPVSTMVK.G
168	595.3064	1188.5982	1188.6139	-13.15	0	63	0.00033	1	U	K.SAETLWNIQK.D
245	646.3586	1290.7027	1290.7183	-12.09	0	83	2.6e-06	1	U	K.QVIQSAYEIIK.L
55	439.2206	1314.6401	1314.6204	14.9	1	45	0.026	1	U	K.LGTDSDKEHWK.N
56	439.2222	1314.6449	1314.6204	18.6	1	(30)	0.77	1	U	K.LGTDSDKEHWK.N
57	439.2307	1314.6704	1314.6204	38.0	1	(22)	5.6	1	U	K.LGTDSDKEHWK.N
58	439.9205	1316.7398	1316.7088	23.5	1	30	0.28	1	U	K.KSAETLWNIQK.D
310	703.8605	1405.7065	1405.7089	-1.70	0	66	8.2e-05	1	U	K.INLNSEEEALFK.K
81	495.5886	1483.7440	1483.7267	11.7	1	67	5.2e-05	1	U	K.ITSGKDYSVSANSR.I
97	512.2765	1533.8078	1533.8038	2.59	1	(14)	28	1	U	K.INLNSEEEALFKK.S
98	512.2845	1533.8318	1533.8038	18.2	1	56	0.0017	1	U	K.INLNSEEEALFKK.S
230	638.9737	1913.8992	1913.8652	17.8	0	43	0.01	1	U	K.GEMMDLQHGSLFFSTSK.I
362	539.7818	2155.0980	2155.0442	25.0	1	16	3.4	1	U	K.LKGEMMDLQHGSLFFSTSK.I

Spot #33
gi|4503987 Mass: 35941 Score: 168 Matches: 6
(1) Sequences: 6(1) emPAI: 0.19
gamma-glutamyl hydrolase precursor [Homo sapiens]
Check to include this hit in error tolerant search

Query	Observed	Mr(expt)	Mr(calc)	ppm	Miss	Score	Expect	Rank	Unique	Peptide
34	397.2071	792.3997	792.3806	24.1	0	18	13	1	U	K.APYEWK.N
148	539.2861	1076.5577	1076.5542	3.24	0	52	0.0048	1	U	R.YYIAASYVK.Y

21 **380.9226 1139.7459 1139.7100 31.5 0 28 0.43 1 U K.KPIIGILMQK.C**
63 **445.9149 1334.7229 1334.6942 21.4 0 29 0.3 1 U K.NLDGISHAPNAVK.T**
92 **501.5905 1501.7497 1501.7354 9.50 0 4 1.1e+02 3 U K.YPVYGVQWHPEK.A**
150 **542.9669 1625.8789 1625.8552 14.6 1 37 0.055 1 U R.LDLTEKDYEILFK.S**

Spot #214
gi|19743875 Mass: 54602 Score: 805 Matches: 25
(9) Sequences: 22(9) emPAI: 1.27
fumarate hydratase, mitochondrial precursor [Homo sapiens]
Check to include this hit in error tolerant search
Query Observed Mr(expt) Mr(calc) ppm Miss Score Expect Rank Unique Peptide
3 **351.1955 700.3765 700.3868 -14.60 0 16 29 1 U K.IANDIR.F**
10 **364.1799 726.3453 726.3371 11.3 0 28 1.1 1 U R.STMNFK.I**
12 **366.2106 730.4067 730.3974 12.7 0 40 0.12 1 U K.IGGVTER.M**
13 **367.2021 732.3897 732.3919 -2.96 0 31 0.82 1 U R.FLGSGPR.S**
14 **367.2352 732.4558 732.3919 87.3 0 (11) 47 3 U R.FLGSGPR.S**
21 **380.7225 759.4305 759.4313 -1.01 0 43 0.043 1 U K.IANAIMK.A**
22 **380.7226 759.4307 759.4313 -0.80 0 (12) 52 5 U K.IANAIMK.A**
29 **393.2337 784.4529 784.4517 1.54 0 27 1.6 1 U R.MPTPVIK.A**
33 **402.7530 803.4915 803.5017 -12.71 1 26 1.1 1 U K.AFGILKR.A**
66 **424.7412 847.4679 847.4803 -14.71 0 31 0.44 1 U K.EFAQIIK.I**
80 **441.7292 881.4439 881.4607 -18.99 0 37 0.091 1 U K.LHDALDAK.S**
99 **479.2453 956.4761 956.4716 4.76 0 60 0.00072 1 U K.YYGAQTVR.S**
6 **355.2165 1062.6276 1062.6073 19.1 1 27 1.1 1 U K.SKEFAQIIK.I**
236 **602.8156 1203.6167 1203.6169 -0.15 0 53 0.0026 1 U R.AIEMLGGELGSK.I**
240 **607.8020 1213.5895 1213.5867 2.34 0 61 0.00035 1 U R.IEYDTFGELK.V**
65 **423.9066 1268.6980 1268.6626 27.9 0 18 4.7 1 U K.IPVHPNDHVNK.S**
357 **745.8580 1489.7014 1489.7049 -2.34 0 52 0.0022 1 U R.AAAEVNQDYGLDPK.I**
363 **749.9079 1497.8013 1497.8555 -36.19 0 59 0.00042 1 U K.VAALTGLPFVTAPNK.F**
114 **500.2988 1497.8745 1497.8555 12.7 0 (23) 2.6 1 U K.VAALTGLPFVTAPNK.F**
118 **504.2712 1509.7917 1509.7576 22.6 1 54 0.0032 1 U K.VPNDKYYGAQTVR.S**
180 **549.6156 1645.8250 1645.8060 11.6 1 12 15 1 U K.RAAAEVNQDYGLDPK.I**
219 **588.6582 1762.9528 1762.9214 17.8 0 56 0.00045 1 U R.IYELAAGGTAVGTGLNTR.I**
220 **589.9584 1766.8535 1766.8727 -10.85 1 42 0.013 1 U R.IEYDTFGELKVPNDK.Y**
341 **709.0730 2124.1972 2124.0772 56.5 0 24 0.77 1 U R.SGLGELILPENEPGSSIMPGK.V**
378 **849.4995 2545.4766 2545.2813 76.7 0 9 34 1 U R.THTQDAVPLTLGQEFSGYVQQVK.Y**

Spot #214
gi|2282013 Mass: 45443 Score: 226 Matches: 11
(0) Sequences: 9(0) emPAI: 0.23
GAPDH-2 like [Homo sapiens]
Check to include this hit in error tolerant search
Query Observed Mr(expt) Mr(calc) ppm Miss Score Expect Rank Unique Peptide
5 **352.1689 702.3233 702.3159 10.5 0 25 2.9 1 U R.YMFSR.D**
9 **357.7361 713.4577 713.4436 19.8 0 32 0.18 1 U R.VVDLLR.Y**
30 **398.2237 794.4328 794.4109 27.6 0 35 0.26 1 K.LTGMAFR.V**
206 **579.8056 1157.5967 1157.6444 -41.27 0 25 1.5 1 U R.LAQPAPYSAIK.E**

207 **581.2825 1160.5504 1160.6190 -59.06 0 33 0.29 1 U K.AGIALNDNFVK.L**
208 **581.8235 1161.6325 1161.6142 15.7 0 30 0.48 1 U R.ELTVGINGFGR.I**
100 **479.2600 1434.7582 1434.7579 0.18 0 21 5.2 1 U R.GAHQNIIPASTGAAK.A**
233 **601.6241 1801.8505 1801.8060 24.7 0 (13) 11 1 U K.LISWYDNEYGYSHR.V**
234 **601.6409 1801.9009 1801.8060 52.7 0 20 2 1 U K.LISWYDNEYGYSHR.V**
162 **534.8030 2135.1831 2135.1779 2.43 0 (2) 2.9e+02 4 U R.EEIKPPPPPLPPHPATPPPK.M**

Spot #557
gi|194377282 Mass: 65889 Score: 673 Matches: 24
(9) Sequences: 20(8) emPAI: 0.88
unnamed protein product [Homo sapiens] Blast searched: result = **pyruvate kinase M1/M2**
Check to include this hit in error tolerant search
Query Observed Mr(expt) Mr(calc) ppm Miss Score Expect Rank Unique Peptide
5 **353.2114 704.4083 704.4221 -19.58 0 14 37 1 K.VFLAQK.M**
7 **353.2203 704.4260 704.4221 5.47 0 (14) 30 1 K.VFLAQK.M**
15 **366.1989 730.3833 730.3861 -3.83 0 21 8.1 1 K.DIQDLK.F**
39 **420.7782 839.5419 839.5341 9.32 0 23 0.8 1 U R.SLLVRPR.S**
66 **434.7449 867.4753 867.4749 0.47 0 30 0.46 1 R.MQHLIAR.E**
71 **453.2632 904.5119 904.5018 11.2 0 41 0.054 1 U K.LFEELVR.A**
84 **490.2603 978.5060 978.4957 10.5 0 56 0.0019 1 R.VNFAMNVGK.A**
87 **495.7594 989.5042 989.5029 1.26 0 50 0.006 1 K.GSGTAEVELK.K**
100 **510.3065 1018.5983 1018.5083 88.4 0 33 0.26 1 K.GDYPLEAVR.M**
108 **517.2711 1032.5276 1032.5968 -67.02 1 17 15 1 U R.KLFEELVR.A**
142 **531.2355 1060.4564 1060.4760 -18.51 0 (10) 54 2 U R.EAEAAMFHR.K**
9 **354.5092 1060.5058 1060.4760 28.1 0 49 0.0042 1 U R.EAEAAMFHR.K**
10 **354.5123 1060.5151 1060.4760 36.9 0 (48) 0.0045 1 U R.EAEAAMFHR.K**
19 **373.5484 1117.6235 1117.5979 22.9 1 36 0.075 1 K.GSGTAEVELKK.G**
196 **571.3087 1140.6028 1140.6026 0.17 0 49 0.0082 1 R.GDLGIEIPAEK.V**
229 **599.3137 1196.6129 1196.6401 -22.70 0 (10) 58 1 R.LDIDSPPITAR.N**
231 **599.3412 1196.6679 1196.6401 23.3 0 35 0.16 1 R.LDIDSPPITAR.N**
318 **731.9021 1461.7896 1461.8079 -12.51 0 49 0.0039 1 K.IYVDDGLISLQVK.Q**
169 **557.9502 1670.8286 1670.7649 38.2 0 54 0.00097 1 U K.GEHYVTGSPTPENQR.T**
216 **589.0125 1764.0156 1763.9781 21.3 1 46 0.0066 1 K.KGVNLPGAAVDLPAVSEK.D**
225 **594.0204 1779.0393 1778.8687 95.9 0 16 5.9 1 K.GADFLVTEVENGGSLGSK.K**
242 **607.9679 1820.8820 1820.9091 -14.86 1 31 0.17 1 R.RFDEILEASDGIMVAR.G**
346 **471.7423 1882.9403 1882.8962 23.4 0 3 94 1 R.LNFSHGTHEYHAETIK.N**
336 **822.4335 2464.2787 2464.2849 -2.55 0 23 1.9 1 R.TATESFASDPILYRPVAVALDTK.G**
